# Identification of Textured Tactile Pictures in Visually Impaired and Blindfolded Sighted Children

**DOI:** 10.3389/fpsyg.2020.00345

**Published:** 2020-03-09

**Authors:** Annie Vinter, Oriana Orlandi, Pascal Morgan

**Affiliations:** LEAD, CNRS UMR 5022, University Bourgogne Franche-Comté, Dijon, France

**Keywords:** blind children, exploratory procedures, tactile pictures, haptic identification, haptic practice

## Abstract

A high level of variability in the capacity of visually impaired children to accurately identify tactile images is reported in the literature, with on average rather low percentages of correct naming responses. However, most of these studies used raised-line drawings as stimuli to be explored and named. The present experiment investigated whether blind children of 3 to 8 years of age would demonstrate a satisfactory ability to name the elements making up tactile images when tested in an experimental setting similar to their natural reading conditions. Textured tactile images taken from genuine illustrated tactile books for young children were used, and the participants received information about the title of the book or listened the text that accompanied each picture before exploration, as it would occur in a natural reading setting. The results showed that their naming scores were indeed higher than previously reported at equivalent ages and did not differ from those of age-matched sighted children. These scores were positively impacted by haptic practice in blind children and correlated with the use of some specific exploratory procedures. The blind children benefited from information provided before exploration, as did their sighted counterparts. However, only in the former did the condition in which full information was provided influence the way the children organized their exploration. The haptic identification scores increased with age regardless of visual status, with the exploration times decreasing in the blind children, while the reverse trend was observed in the sighted children. These results are discussed at the light of the image-mediation model of haptics, suggesting that during the age period considered in the present experiment, blind children would progressively learn to process haptic information directly, thus leading to a decrease of exploration times, while sighted children would learn to translate haptic information into a visual image used to retrieve semantic information, involving an increase of their exploration times.

## Introduction

Originally carried out by a few groups of scientists (e.g., Heller, 1989; Lederman et al., 1990; D’Anguilli et al., 1998), research dedicated to the haptic (i.e., the sense of active touch, Revesz, 1950) identification of two-dimensional (2D) pictures has become the object of increasing interest in recent years, as testified to by recent publications (Mazella et al., 2018; Overvliet and Krampe, 2018; Lebaz and Picard, 2019; Vinter et al., 2019). If recognizing common 3D objects on the basis of haptic information alone is a highly efficient daily activity for children irrespective of their visual status (Simpkins, 1979; Bushnell and Baxt, 1999), identifying what 2D haptic patterns depict is a less frequent activity for sighted persons, but not for visually impaired individuals who read Braille patterns or interpret tactile maps or diagrams or images in tactile books, etc. One exception concerns the illustrated books intended for very young sighted children. These often contain tactile pictures that are very attractive to them and elicit active interactions through explorations and manipulations. Being able to identify by touch what tactile patterns depict is therefore a relevant challenge that most young children face.

A large number of studies in this domain have used raised-line drawings as stimuli to be explored and named (that is, contour-line drawings of approximately 0.5 mm in height produced on heat-sensitive paper or with a pen tracing on a special plastic film fixed on a rubber board). A high level of variability in the reported correct naming scores can be found throughout these studies which, however, agree in acknowledging that this is “a hard but not impossible task,” to quote Picard and Lebaz (2012). Covering 16 different studies performed with children or adults, this review makes it clear that the percentages of correctly identified pictures ranged from 9 to 85%, with a mean value of 42%. Several factors certainly account for this considerable variation, in particular the visual status and age of the participants, the quantity of semantic information given to them before exploration, their level of haptic practice or still, the type of exploratory procedures they used when exploring the tactile images.

Regarding visual status, no clear image emerges from the results reported in different studies concerning correct naming responses for 2D tactile pictures (see Cattaneo and Vecchi, 2011, for a general review comparing performance of early or late blind individuals with sighted persons in different spatial tasks). To simplify the results somewhat, some studies concluded that visually impaired individuals perform better than blindfolded sighted ones (Heller, 1989; D’Anguilli et al., 1998; Mazella et al., 2014; see Pathak and Pring, 1989, for a similar conclusion in a matching recognition task), others that blindfolded sighted participants outperform visually impaired ones (Lederman et al., 1990; Heller et al., 1996), while yet others have failed to observe any significant differences as a function of visual status (Mazella et al., 2016). However, this overview becomes even more complex when we consider experiments which have distinguished between late blind participants and early blind persons, with the former systematically exhibiting better performance than the latter and even outperforming the sighted participants (Heller, 1989; Heller et al., 2003). Late blind individuals are indeed likely to have been confronted with 2D visual representations in the past and to know the “rules” of graphic representations (Willats, 2005), thus giving them an indisputable advantage over early blind people. Interestingly, sighted individuals have been reported to be better in tactile image identification than visually impaired individuals in an experiment in which no textured tactile images were used, whereas no difference was observed in the case of textured tactile images (Thompson et al., 2006). This latter result was due to a noticeable improvement in the performance of the visually impaired group. This finding echoes the results reported by Theurel et al. (2013), who demonstrated that tactile images made of textured pictures are identified better by early blind children than thermoformed or raised-line pictures.

The few studies that have investigated the effect of age on the identification of tactile pictures have indicated that this ability develops gradually. Three age groups were included in the Picard et al. (2013) which was carried out with blindfolded sighted individuals who were asked to identify raised-line drawings. The percentage of correct naming responses was 33% at 5–7 years of age and reached 69% at 13–17 years of age and 86.5% in adults. A more detailed view of this development was provided in Mazella et al. (2016), who studied 6 age groups from childhood (5–6 years of age) to adulthood (18–25 years of age) and examined both visually impaired and sighted individuals. Although picture identification scores for raised-line drawings increased with age regardless of visual status, the improvement was quite small between 5 and 6 years of age and 9 and 10 years of age. This finding was confirmed by Overvliet and Krampe (2018) who reported a very gradual increase in performance between 4 and 5 years of age and 10–12 years of age in blindfolded sighted participants in a task using large raised-line drawings. Interestingly, this study indicated that exploration times for accurate haptic identification significantly increased over this period. The authors suggested that due to their less efficient working memory, younger children were inclined to guess what each image depicted, while older children were able to gather and process more information, spending more time on exploration before naming the pictures.

The ability to name tactile pictures has been shown to vary as a function of the semantic information given to the participants prior to or after haptic exploration (Heller, 1989; Pathak and Pring, 1989). Heller et al. (1996) reported higher identification scores, together with faster response times, in blindfolded adults who were provided with prior category information about the tactile pictures than in participants who were only told that the pictures depicted objects that could be named. These results did not change when the category information was provided after exploration, just before the identification of the pictures. The authors argued that this effect of providing category information was likely to influence the identification or naming processes, and especially the retrieval of picture names in semantic memory, rather than the perceptual processes involved in the exploration phase, since the effect persisted when the information was given after exploration. Picard et al. (2014) investigated the role played by this contextual factor in the ability to name pictures and examined how it interacted with practice. To this end, they made a comparison with sighted children who lacked practice with this type of picture. In their experiment, blindfolded sighted and visually impaired children of 9–10 years of age (early blind and low vision), who knew the category of the depicted objects, had to name raised-line drawings. The children with early visual impairments were found to perform the best, in terms of both accuracy and response times. The authors claimed that the provided information enabled the children to make hypotheses about the object’s identity, thereby guiding their exploration better, a claim opposite to that made by Heller et al. (1996). They suggested that the more efficient procedures deployed by visually impaired children enable them to outperform their sighted counterparts.

The present experiment aimed at investigating whether early blind children would be able to identify tactile images better than reported in the literature when the experimental setting was designed to be as similar as possible to their natural ecological reading conditions. For this scope, following Theurel et al. (2013), we used textured tactile pictures taken from genuine illustrated tactile books intended for young visually impaired children. This technique makes the different elements or parts composing the depicted object (i.e., the roof, the windows, etc… for a house, or the head, the legs, etc… for the human figure) easier to distinguish from each other, as they are made of distinct textures in relief, while simultaneously providing information about their shape, with the borders of each textured area delineating the element’s contours. This should enhance the identification process in blind children, possibly leading them to outperform age-matched sighted counterparts. The present experiment included two young age groups (3–6 years of age, 6–8 years of age). Given that the use of textured tactile images enhances haptic identification performance much more than raised-line drawings, we expected to observe a significant age effect already between 3–6 and 6–8 years of age in the sighted as well as in the blind group, contrary to what is reported in the current literature. Furthermore, the issue of the role played by prior semantic information was operationalized by contrasting two conditions: one in which the participants received information about the title of the book from which the tactile images were taken (this condition was similar to providing category information) and another in which the experimenter read them the text that accompanied each picture before exploration (full information), in the same way as it would occur in a natural reading setting. The children were asked to identify each textured element present in the image, a task that required them to parse the whole image into its components or parts. This task demanded more analytical processing than had been elicited in earlier studies. Might identification performance be facilitated in young blind children when the experimental conditions better mimic their natural reading conditions? We expected the quantity of prior semantic information provided to have a significant, but broadly similar, impact on the ability of both blind and sighted children to correctly name the elements present in the images: while the former might benefit from their familiarity with tactile images and stronger analytical functioning bias (Puspitawati et al., 2014), the latter have a greater knowledge of the graphic rules used for drawing 2D representations of objects, especially in the case of the two topics chosen for our task (house and human figure).

Finally, the influence of two others variables was investigated in the present experiment. Although the impact of haptic practice on tactile image identification has been emphasized in several studies (D’Anguilli et al., 1998; Picard et al., 2014), none of them has directly tested whether visually impaired children with an important amount of practice perform better than those with less practice. The role of practice in individuals suffering from visual deficits has been revealed in experiments dealing with various spatial skills (e.g., Dulin and Hatwell, 2006; Ittyerah, 2009). A significant learning effect across trials has also been demonstrated in a haptic spatial matching task in visually impaired as well as in sighted adults (Postma et al., 2007). More relevant to our aim here is the fact that a positive effect of practice has been reported on drawing ability in blind individuals, i.e., their ability to produce 2D images (D’Anguilli and Maggi, 2003). For instance, Vinter et al. (2018) showed that the more visually impaired children aged 6–14 years practised drawing, the more recognizable their drawings were and the more elements they contained. In the present experiment, we asked the parents to code the level of illustrated tactile book practice and of Braille practice of their blind child. In line with the previous results, we expected to observe a positive significant correlation between haptic practice and identification performance in children with vision loss.

The last factor that was of interest to us in the present experiment was related to the role played by the procedures used by children during haptic exploration of the tactile pictures. This issue raises the question of whether the way information is collected through exploratory procedures contributes to the accuracy of perceptual outcomes: in our case, identification responses. Magee and Kennedy (1980) reported that inexperienced blindfolded sighted adults were more accurate in their recognition when they were passively guided during their exploration of raised-line displays. This finding has been confirmed in blindfolded sighted children, who achieved similar performance to blind children when they were passively guided, but poorer performance when they were active (D’Anguilli et al., 1998; D’Anguilli and Kennedy, 2000). According to the authors, this result demonstrates that the blind children had better exploration skills, an advantage that was eliminated when the sighted children were placed in the guided exploration condition. More direct relations between modalities of exploration and identification performance have been reported in adults. Klatzky et al. (1993) showed that recognition accuracy improved when five fingers were used rather than only one. Moreover, exploring raised-line drawings with two hands instead of one improved identification performance in blindfolded sighted adults (Wijntjes et al., 2008a, b). With regard to children, in a joint tactile book reading setting, Bara (2014) reported that the explorations of five visually impaired children between 5 and 8 years of age were more often unimanual than bimanual, and that children employed the contour following procedure more often when they had to guess the meaning of tactile images than to associate meaning and images. To date, no experimental study has investigated this issue comparing blind and sighted children (see, however, Vinter et al., 2012, for a study investigating the links between exploratory procedures and the production of raised-line drawings). In the present experiment, we wanted to assess the correlations between the modalities of manual exploration and correct identifications of the elements present in the tactile pictures. We expected that bimanual exploration would facilitate haptic identification and, more specifically, that the use of the contour following procedure, which is specific to the extraction of the shape property (Lederman and Klatzky, 1993), should correlate with the production of correct naming responses. Furthermore, Picard et al. (2014) reported a significant negative correlation between response times and correct naming in participants with vision loss. They claimed that the more efficient exploratory procedures used by the latter could account for the faster location, during exploration, of confirmatory cues relating to the identity of the tactile picture that had already been partially induced by the available semantic information. In line with this study, we expected to observe shorter exploration times in the blind children than in their sighted counterparts.

## Materials and Methods

### Participants

Seventy-two children between 3 and 8 years of age participated in the study. They were divided into two groups according to their visual status. One group was composed of 32 early totally or legally blind children (18 totally blind children did not perceive light, WHO – World Health Organization – category: 5; 4 totally blind children had minimal light perception, WHO category 4; and 10 legally blind children had profound vision loss with a visual acuity inferior to 1/20, WHO category 3). Eighteen of these children were 3 to 6 years of age (*M* = 4 years;6 months, *SD* = 0.9) and 14 were 6 to 8 years of age (*M* = 6 years;9 months, *SD* = 0.85). The second group consisted of 40 sighted children, with 20 of them being aged 3 to 6 years (*M* = 4 years;5 months, *SD* = 0.80) and 20 aged 6 to 8 years (*M* = 7 years, *SD* = 0.9). The sighted children were typically developing children with normal or corrected to normal vision. Table 1 provides additional information about the participants.

**TABLE 1:** Main characteristics of the groups of children involved in the study.

	Age group	Number	Sex *N*	Mean age and range (years;months)	WHO visual category (*N* per category)	Braille practice (*N* per level)	Tactile book reading practice (*N* per level)
Group with visually impaired children	3–6 years	18	10 girls 8 boys	*M* = 4;6 (3;1 to 5;9)	C3 = 6 C4 = 2 C5 = 11	L1 = 8 L2 = 2 L3 = 4 L5 = 4	L1 = 3 L2 = 8 L4 = 5 L5 = 2
	6–8 years	14	6 girls 8 boys	*M* = 6;9 (6;2 to 8;1)	C3 = 4 C4 = 2 C5 = 8	L3 = 4 L5 = 10	L1 = 2 L2 = 4 L3 = 2 L4 = 4 L5 = 2
Group with sighted children	3–6 years	20	10 girls 10 boys	*M* = 4;5 (3;8 to 5;11)			
	6–8 years	20	14 girls 6 boys	*M* = 7 (6;0 to 7;11)			

*WHO, world health organization categories: C3, profound vision loss; C4, minimal light perception; C5, no light perception; Braille or Tactile book reading Practice: L1, no practice at all; L2, rare practice; L3, 1 or 2 times a month; L4, 1 or 2 times a week; L5, almost daily practice.*

The visually impaired children did not present any associated psychiatric or cognitive disorders and attended the appropriate school grade for their chronological age. The etiologies of the visual impairments were known for 30 of them. These took the form of cataracts (*n* = 2), Leber’s congenital amaurosis (*n* = 4), glaucoma (*n* = 2), retinopathy of prematurity (*n* = 4), optic nerve atrophy (*n* = 4), amblyopia (*n* = 3), microphthalmia (*n* = 3), severe myopia (*n* = 4), retinitis pigmentosa (*n* = 2), and retinoblastoma (*n* = 2). Their parents were asked to code the regularity of their practice in Braille and tactile book reading on a 5-point scale (level 1 = no practice at all, level 2 = infrequent practice, level 3 = 1 or 2 times a month, level 4 = 1 or 2 times a week, level 5 = almost daily practice).

We checked with their parents that none of the blind children were familiar with the images contained in the two illustrated tactile books used in this experiment (none of them had these books at home); the sighted children were unfamiliar with any tactile images. Whether visually impaired or not, the children were tested individually at school. They mostly came from middle class families. Informed written consent was obtained from their parents. The experiment was conducted in accordance with the tenets of the World Medical Association Declaration of Helsinki on Ethical Principles for Medical Research Involving Human Subjects.

### Material

**FIGURE 1:**
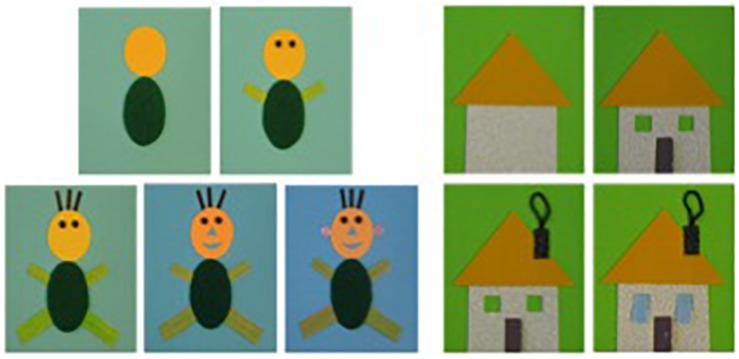
Illustration of the tactile pictures explored by the children: the human figure series on the left, the house series on the right.

The tactile images were adaptations of pictures taken from genuine tactile books for young children and produced by a publisher of tactile illustrated books (Les Doigts Qui Rêvent- Dreaming Fingers). One series of images was related to the topic of the human figure (taken from “Petitpoint dessine un bonhomme” – Smallpoint draws a man) and contained five images; the other comprised five images and was related to the topic of houses (taken from “Petitpoint bâtit une maison” – Smallpoint builds a house). These two books are popular among young visually impaired children, as attested to by the publisher, and deal with highly familiar contents. However, we were not interested in the possible differences engendered by these two topics (man and house). The dimensions of the images were 15 × 15 cm. Figure 1 shows the types of elements that were present in each tactile picture of the two series (see also Table 2).

**TABLE 2:** Titles and texts accompanying each image in the two series of tactile images (elements are underlined).

Title: “This is the story of Smallpoint who builds a house”.
Image 1: “I put a big rectangle at the bottom of the picture to depict the walls of the house, and I stick on a big triangle to form the roof”.
Image 2: “I put a small rectangle on the wall to make the door, and in the middle of the wall, two small squares for the windows”.
Image 3: “On the roof, a small rectangle makes the chimney and I also put on wool to imitate the smoke coming out of the chimney”.
Image 4: “I add fabric to the windows to represent the curtains, and a handle on the door to enter my house”.

Title: “This is the story of Smallpoint who draws a man”
Image 1: “At the top of the picture, I put a circle for the head, and then a big oval under the head for the belly”.
Image 2: “On the circle, I stick two small circles for the eyes, and two short, thin rectangles on each side of the oval for the arms”.
Image 3: “Above the circle, I fix some sticks for the hair. And two long, thin rectangles at the bottom of the oval for the legs”.
Image 4: “I draw a triangle under the eyes for the nose, and a half-moon under the nose for the mouth”.
Image 5: “Finally, two circles on each side of the face will be the ears”.

**FIGURE 2:**
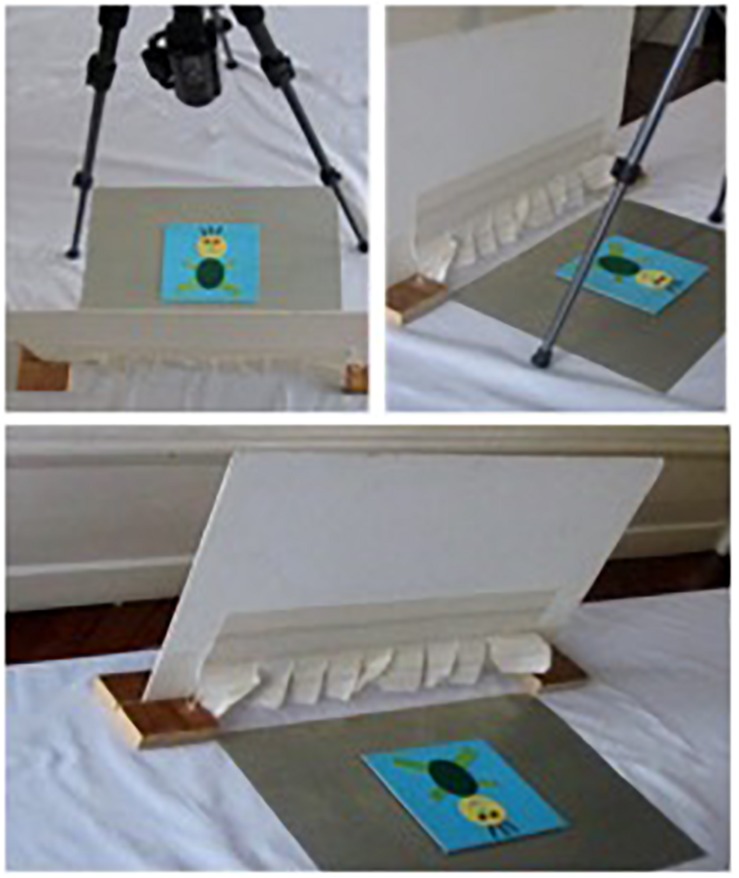
Views of the experimental setup.

The images consisted of elements in relief with different shapes and textures (for instance, the roof of the house was a triangle made from corrugated cardboard, the head of the man was a circle in felt). As illustrated in Figure 1, the first image of each series contains two elements, and two new elements are added in each of the following images, except in the fifth image of the house series, where only 1 new element is included. To place the children, regardless of their visual status, in a condition of blindness during exploration, a vertical screen of 50 cm in width by 32 cm in height was placed on the table, with the images being located behind it, as illustrated in Figure 2. The screen had an opening (32 cm × 6 cm) at the bottom, through which the children were asked to pass their hands in order to explore the images. The images were held in a fixed position using a system of magnets. As shown in Figure 2, a camcorder was positioned above the tactile images in order to record the precise details of the children’s explorations.

### Procedure

The children were comfortably seated in front of the experimental device with the experimenter at their side. During an initial familiarization phase, they were presented (visually and/or haptically) with tactile images of the same size as those used in the experiment but taken from other books. The experimenter showed the children how to explore the images by moving their hands over the surface and following contours with their fingers in order to identify the textured elements that composed the images. However, note that this phase was not a learning phase aimed at teaching children how to deploy exploratory procedures, but a simple demonstration phase that also helped to reassure young sighted children put in an unfamiliar situation of touching without seeing. The children were told that they would later have to explore similar images as accurately as possible and to indicate “what they identified in the picture,” “what they could recognize,” “what the explored elements depicted,” “what the elements contained in the picture represented,” by placing their fingers on the element in question and saying what it represented. Then, in the second part of the familiarization phase, the experimenter placed one image on the table, behind the screen, and showed the children how to pass their hands through the opening in order to explore it. The children were asked to perform this sequence of movements several times – passing the hands through the opening, exploring the image, moving their fingers over the elements present on the image and trying to report what they recognized. When the children did not move their hands and fingers over the entire surface of the image, the experimenter guided the children’s movements so that they touched every part of the image. This encouraged them to be active in their explorations and familiarized them with the image size.

The experimental phase started when the children’s explorations and identifications (whether correct or not) showed that they understood the task. The visually impaired and sighted children were divided into two groups according to the type of semantic information that was given before the exploration of each image. In one group (partial information, *n* = 20 sighted children or *n* = 16 blind children), the participants were informed of the title of the book from which the images were taken. Thus, after having placed the first image of the “man” series behind the screen, for instance, and before the children passed their hands through the opening, the experimenter told them that they would have to explore images that were taken from a book entitled “Smallpoint draws a man.” They were systematically reminded of the title of the story before exploring each picture of the series. In the second group (full information, *n* = 20 sighted children or *n* = 16 blind children), the experimenter announced the title of the book and then read the written text that accompanied each image before the exploration started. These texts were taken from the original tactile books. For instance, in the case of the house series, the experimenter read the following text, before the children started exploring the first image: “I put a big rectangle at the bottom of the picture to depict the walls of the house, and I stick on a big triangle to form the roof.” Table 2 presents these texts. Whatever the series, the texts systematically enumerated and localized the new elements that had been added to the image as compared to the previous ones (the elements are underlined in the texts shown in Table 2). Geometrical cues and, in one case, a texture cue were given.

Once the prior information had been provided, the children passed their hands through the opening and were free to explore the image for as long as they wanted. They were reminded to mention all the elements they could recognize by putting their fingers on each identified element. When they considered that they had finished exploring the picture, they withdrew their hands from it and put them in front of the screen until the experimenter gave them the relevant information about the next image and asked them to explore it. In each condition (partial or full information), half of the children explored the man series and half the house series. The computed identification scores (see below) did not take account of the content of the series of images given to the children. This variable was not of interest in the present experiment.

The experimenter did not give any feedback about the identifications made by the children and encouraged them to explore each image as accurately as possible. The experimental phase was video-recorded, with a close-up focus on the children’s hands and fingers (see Figure 2).

### Coding of the Data

The offline analysis of the video-recordings formed the basis for the coding of a number of variables by two pairs of two judges working independently. In the present manuscript, we focus on the variables described below, which relate to the correct identifications of the explored elements and some of the exploratory procedures used by the children.

#### Correct Semantic Identifications

A correct semantic identification was coded each time the children simultaneously touched an element of the image with their fingers and named it correctly. When they simply said the name of an element present in the image, the experimenter systematically asked them to locate it precisely with their fingers. A correct semantic identification was only coded if both the indicated location and verbal identification were correct. For each image and child, the number of correct semantic identifications was divided by the total number of elements present in the image, and a global correct semantic identification score was computed by summing the proportion of correct semantic identifications in each image and dividing this by the number of images present in the series (4 or 5).

#### Correct Geometrical Identifications

A correct geometrical identification was coded each time the children touched an element of the picture with their fingers and correctly reported its geometrical shape (for instance, while touching the roof in the house series, a correct geometrical identification was noted when the children identified a triangle).

#### Correct Texture Identifications

A correct texture identification was coded each time the children touched an element of the picture with their fingers and correctly reported its texture (for instance, while touching the roof in the house series, a correct texture identification was noted when the children said that this was a piece of cardboard).

Geometrical and texture identification scores were computed for each child using the same procedure as that described previously for the semantic identification scores. It should be noted that these responses were not exclusive: the children could both identify the elements explored correctly (“here is the roof” for instance) and add that this element “is a triangle”, and/or that “it is made of cardboard”.

#### Exploratory Procedures

The *exploratory procedures* used by the children for each tactile image were coded by four previously trained judges working independently, in the same way as described in Vinter et al. (2012). They coded the occurrences of different procedures, of which only the results of two are reported, those that gave rise to a hypothesis presented in introduction (bimanual exploration, contour following procedure), completed by a specific modality of execution of these procedures (use of symmetrical movements):

##### Bimanual exploration

Deployment of active exploratory movements of the two hands working simultaneously (see Davidson, 1972);

##### Contour following procedure

Coded in the case of dynamic edge following using finger movements (see Lederman and Klatzky, 1987, for a more extensive definition);

##### Symmetrical movements

Coded in the case of deployment of symmetrical finger movements around the shapes regardless of the procedure used (see Ballasteros et al., 1997, for a more extensive definition).

Finally, the *mean time of exploration* (in sec) of the tactile images was also computed by averaging each child’s individual exploration times for the different images.

The 324 sequences to be coded (72 children exploring either 4 or 5 tactile images) were randomly divided into two subsets corresponding to the data of 36 children each and were, respectively, assigned to the two pairs of two judges, who were unaware of the visual status and age of the children they had to analyze. The mean percentages of interjudge agreements were 91.3% for the correct identifications and 87% for the exploratory procedures for one pair of judges and 92.6 and 82%, respectively, for the other pair. All disagreements were settled by the two pairs of judges working together before analysis.

Non-parametric tests were mainly used for the statistical analysis because homoscedasticity did not apply in most cases. The Mann-Whitney test (*U* value) was employed to test differences resulting from Visual Status (visually impaired versus blindfolded sighted), Age (3–5 years, 6–8 years) and Prior information (partial versus full information) in the percentage of correct semantic, geometrical and texture identifications. The Kendall test (*τ* value) was used to compute correlations between the degree of tactile image or Braille practice and the frequency of occurrences of the different types of identifications in the visually impaired children. Correlations (*τ* value) were also computed between the percentages of use of specific exploratory procedures and the production of semantic, geometrical or texture identifications in the two groups of children. Finally, as the distribution of the exploration times did not deviate significantly from normality (*p* > 0.50), an ANOVA with Age group, Visual Status and Prior information was run with the exploration times as the dependent variable.

## Results

**FIGURE 3:**
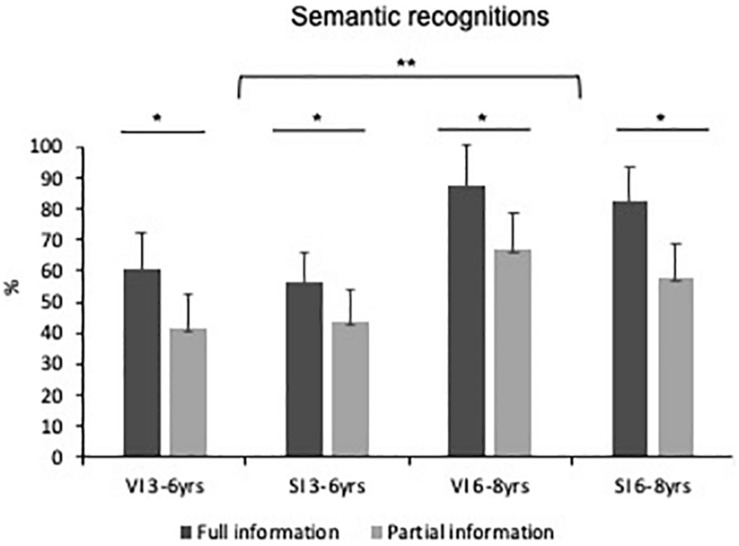
Frequency of occurrence of semantic identifications as a function of age, visual status (VI, visually impaired children; SI, blindfolded sighted children) and type of prior information. The values given are means with standard error bars. *indicates significance with *p* < 0.05; **indicates significance with *p* < 0.01.

Figure 3 shows the percentages of correct semantic identifications as a function of age, visual status and type of prior information.

As illustrated in Figure 3, the children aged 6 to 8 years produced significantly more correct semantic identifications (*M* = 73.1%, *SD* = 30%) than the younger children (*M* = 50.5%, *SD* = 33%), regardless of visual status and type of prior information (*U* = 383.5, N1 = 38, N2 = 34, *p* < 0.01). This age effect remained significant irrespective of whether they received full information or partial information prior to exploration (*p*_s_ < 0.05), and of whether the children were sighted or not (*p_s_* < 0.05). Visual status did not reach significance: visually impaired children (*M* = 62.5%, *SD* = 36%) performed as well as blindfolded sighted children (*M* = 60%, *SD* = 36%; *U* = 595.5, N1 = 32, N2 = 40, *p* = 0.61). The children who benefited from full prior information were better (*M* = 70.7%, *SD* = 31%) at identifying the elements contained in the tactile images than those who explored the images knowing only the story’s title (*M* = 51.6%, *SD* = 34%; *U* = 416, N1 = 36, N2 = 36, *p* < 0.01). This was observed in the blind group (*p* < 0.05), as well as in the sighted group (*p* < 0.05).

Furthermore, we looked at the correlations between haptic practice and the production of correct naming responses in the 32 children with vision loss. Table 3 shows that the higher the level of regularity of Braille practice, the higher the percentage of correct semantic identifications (*τ* = 0.53, *p* < 0.01). The correlation with the level of regularity of tactile image practice was also positive, but marginally significant (*τ* = 0.22, *p* = 0.08).

**TABLE 3:** Correlations (*τ* values) between the production of correct naming, geometrical and texture responses and haptic practice (Braille, tactile image) or mean exploration times in the blind children.

	Naming	Geometrical	Texture
	responses	responses	responses
Braille practice	0.53**	0.28*	0.01
Tactile image practice	0.22	−0.26*	–0.18
Mean exploration times	−0.37**	–0.10	0.17

*Indicates significance with *p* < 0.05; **Indicates significance with *p* < 0.01.

Table 4 reports the correlations between the use of specific exploratory procedures and the production of correct semantic identifications. The more the visually impaired children employed the contour following procedure (*τ* = 0.54, *p* < 0.01), or the bimanual procedure (*τ* = 0.38, *p* < 0.01), or used symmetrical movements (*τ* = 0.36, *p* < 0.01), the more they correctly identified the elements contained in the tactile images. The results for the blindfolded sighted children showed that the more they employed the contour following procedure (*τ* = 0.41, *p* < 0.01), the more they correctly named the elements in the pictures.

**TABLE 4:** Correlations (*τ* values) between the production of different types of identification responses or the mean exploration times, and the use of specific exploratory procedures in the blind and sighted children.

	Naming responses		Geometrical responses		Texture responses		Mean exploration times	
				
	Blind	Sighted	Blind	Sighted	Blind	Sighted	Blind	Sighted
Bimanual exploration	0.38**	0.14	0.16	–0.04	–0.17	–0.03	−0.38**	0.01
Contour following procedure	0.54**	0.41*	0.31*	0.33*	0.12	–0.15	–0.10	0.09
Symmetrical movements	0.36**	0.16	0.27*	–0.06	–0.17	–0.20	−0.35**	0.02

*Indicates significance with *p* < 0.05; **Indicates significance with *p* < 0.01).

**FIGURE 4:**
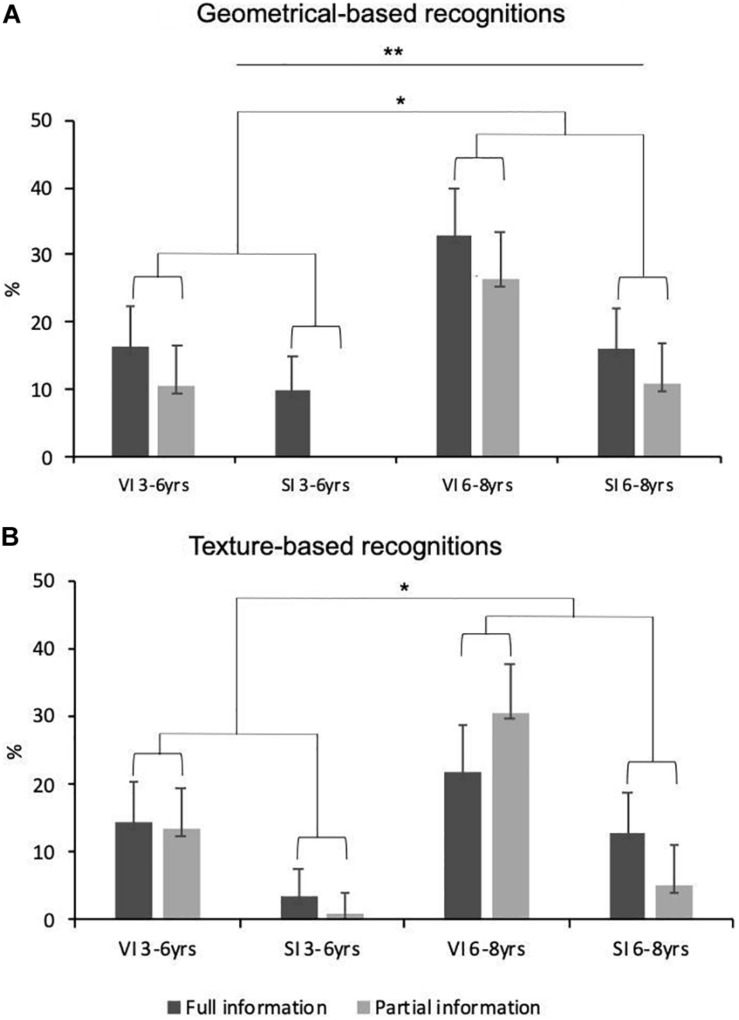
Frequency of occurrence of geometry-based **(A)** and texture-based **(B)** identifications as a function of age, visual status (VI, visually impaired children; SI, blindfolded sighted children) and type of prior information. The values given are means with standard error bars.

When asked to indicate everything they could identify in the explored pictures, the children sometimes reported correct geometrical or texture identifications. Figure 4 (A: geometrical-based recognitions; B: texture-based recognitions) presents these data as a function of visual status, age and prior information.

Visual status reached significance: children with visual impairments mentioned the textures (*M* = 19.2%, *SD* = 25%) as well as the shapes (*M* = 20.5%, *SD* = 20%) of the explored elements more often than the blindfolded sighted children (*M* = 5.6%, *SD* = 11% and *M* = 9.2%, *SD* = 16%, respectively; *U* = 429.5 and *U* = 413.5, respectively, N1 = 32, N2 = 40, *p*_s_ < 0.05). The occurrence of geometrical identifications increased significantly with age (*M* = 8.9%, *SD* = 15% in the 3–6 year-olds, *M* = 20%, *SD* = 21% in the 6–8 year-olds; *U* = 420, N1 = 38, N2 = 34, *p* < 0.01). This age effect was marginally significant for the texture-related responses (*U* = 495, N1 = 38, N2 = 34, *p* = 0.06), the frequency of which increased between 3–6 years of age (*M* = 7.8%, *SD* = 15%) and 6–8 years of age (*M* = 16%, *SD* = 23%). Finally, as Figure 4 makes clear, benefiting from partial or full prior information did not impact the production of texture identifications (*p* > 0.80), or of geometrical identifications (*p* > 0.15), even though these later responses were a little more frequent in the full information (*M* = 17.6%, *SD* = 20%) than in the partial information condition (*M* = 10.7%, *SD* = 17%). Differentiating between the blind group and the sighted group did not reveal any significant effect of this factor (*p*_s_ > 0.20). As shown by Table 3, Braille or tactile image practice did not influence the production of correct texture identifications in the visually impaired children (*p_s_* > 0.20). By contrast, the correlations between haptic practice and correct geometrical responses yielded interesting findings. On the one hand, the greater the level of regularity of tactile image practice, the lower the production of correct geometrical identifications (*τ* = −0.26, *p* = 0.05). On the other, the higher the level of regularity of Braille practice, the greater the production of correct geometrical identifications (*τ* = 0.28, *p* < 0.05).

Significant correlations emerged when the exploratory procedures and the production of correct geometrical identifications were crossed (see Table 4). None reached significance when the correct texture identifications scores were considered. In the visually impaired group, correct geometrical identifications increased when the children used symmetrical movements (*τ* = 0.27, *p* = 0.04), or the contour following procedure (*τ* = 0.31, *p* = 0.02). The latter correlation also reached significance in the blindfolded sighted group (*τ* = 0.33, *p* = 0.01). In addition, it is worth noting that the blind children used the contour following procedure significantly more often in the full information condition (*M* = 12.4%, *SD* = 14.4%) than in the partial information condition (*M* = 3.3%, *SD* = 7.2%; *U* = 83, N1 = 16, N2 = 16, *p* < 0.05). There was no similar impact of the quantity of prior semantic information in the sighted group (*p* > 0.20).

**FIGURE 5:**
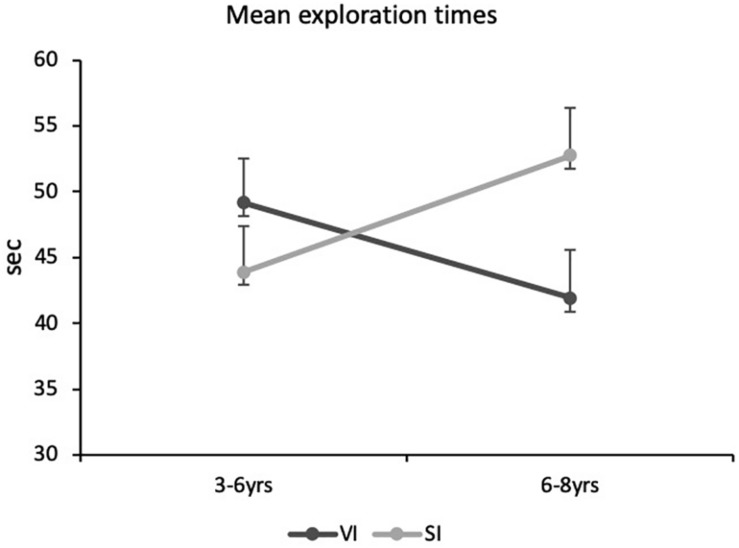
Mean exploration times of the tactile images as a function of age and visual status (VI, visually impaired children; SI, blindfolded sighted children). The values given are means with standard error bars.

Finally, the mean exploration times were analyzed with an Anova carried out with Age Group, Visual Status and Prior Information as between-subjects factors. Only the interaction between age group and visual status reached significance [*F*(1,64) = 4.9, *p* = 0.03, *n*^2^ = 0.07]. Figure 5 depicts this interaction.

Figure 5 shows that the mean times increased as a function of age in the blindfolded sighted children, while they tended to decrease with age in the visually impaired children. A Fisher’s LSD *post hoc* test revealed that, on average, the older blindfolded sighted children spent significantly less time exploring the tactile images than the age-matched visually impaired children (*p* < 0.05). Interestingly, in the visually impaired children (see Tables 3, 4), the shorter the mean exploration times, the more frequently they produced correct semantic identifications (*τ* = −0.37, *p* < 0.01), used bimanual explorations (*τ* = −0.38, *p* < 0.01) or symmetrical movements (*τ* = −0.35, *p* < 0.01). No correlation involving exploration times reached significance in the blindfolded sighted children (*p*_s_ > 0.10). By way of an example, we can point to the correlation between exploration times and correct naming responses in these children (*τ* = 0.10, *p* > 0.30).

## Discussion

The present experiment tested whether young blind children would be able to identify tactile pictures better than is reported in the literature when the experimental setting was designed to be as similar as possible to their natural tactile reading conditions. The average percentages of correct naming of raised-line drawings reported in the past have barely exceeded 35% in children aged less than 9 years (Mazella et al., 2016; Overvliet and Krampe, 2018). To create such conditions, we used textured tactile pictures taken from popular illustrated tactile books intended for visually impaired children, accompanied by the texts used in these books. This condition, in which full information was provided to the children before they explored the images, was contrasted with a condition in which the participants received only partial information by being told the title of the book from which the images had been taken. The influence of age and of visual status was considered in the present experiment. Furthermore, this study was the first to additionally assess both the role played by blind children’s haptic practice and exploratory procedures on their capacity to correctly identify elements contained in the tactile images.

As expected, naming performance reached a higher level in the present study than has previously been observed in studies involving children aged less than 9 years, with an overall mean of 50% correctly named elements in the partial information condition. This is close to that obtained when prior categorical information is provided, as has frequently been the case in previous studies (see, for instance, Picard et al., 2013). The main reason for this better performance is certainly related to the use of textured images rather than raised-line drawings, as suggested by Theurel et al. (2013). Thompson et al. (2003) claimed that texture delineates regions corresponding to elements or parts of the overall image, acting as a kind of “uniform connectedness” (Palmer and Rock, 1994) that simplifies the identification of the object’s elements or parts. The way objects are depicted in tactile images influences the efficiency of the recognition processes (Kalia and Sinha, 2011), and our results suggest that individualizing the object’s elements by means of textures has a positive influence. The importance for blind children of constitutive elements in the representation of objects was indeed revealed in the drawing study conducted by Vinter et al. (2018), which found that blind children tended to draw familiar objects element by element, juxtaposed with one another but spatially sufficiently well integrated relative to one another to enable an acceptable level of recognition.

With regard to the production of correct semantic identifications, our results did not indicate any significant differences in performance between blind and sighted children, with the overall success rate reaching 60–62%. Similarity of performance between blind and sighted children has already been reported in Mazella et al. (2016), where prior category information was provided to the participants, as well as in Thompson et al. (2006), who asked adults to identify textured tactile images. As pointed out in the introduction, other studies have concluded both that blind participants outperform sighted ones and vice versa (Heller, 1989; Lederman et al., 1990). In our opinion, the great variability in these results shows that the answer to this issue is highly dependent on several contextual factors, whether internal to the participant (such as, for instance, those evoked in the present experiment, i.e., age, prior knowledge, practice, quality of exploration) or external (in particular, the properties of the tactile images used). It should also be recalled that our study did not include late blind children or children with low vision, i.e., visually impaired children who could make use of knowledge acquired in the past or acquired even through degraded vision in addition to their ability to process haptic information. Indeed, numerous findings show that individuals with low vision outperform blindfolded sighted participants in spatial tasks (see Cattaneo and Vecchi, 2011, for a review).

However, this may be, our results support Kennedy (1993) that people deprived of vision can accurately process 2D spatial information about objects at a similar level as sighted individuals, in our case by making use of a combination of information based on contours and textures. In a series of experiments, Desmarais et al. (2017) consistently demonstrated that haptic and visual object identification are based on similar processes which may possibly share memory representations that contain shape information.

Furthermore, regardless of visual status, the children in both groups were more successful in naming the elements contained in the tactile pictures when they used suitable exploratory procedures, and more specifically the contour following procedure, which is best suited to extracting shape information (Lederman and Klatzky, 1987, 1993). In blind children, this relationship was also observed in the case of the use of the bimanual procedure and symmetrical movements. In line with this observation, the current literature also strongly suggests that bimanuality plays a special role in blind participants. For instance, when exploring with both hands, blind adults were found to be better than sighted participants at discriminating elliptic shapes (Davidson, 1972; Russier, 1999). In a spatial memory task, Leo et al. (2018) reported that performance was positively correlated with the use of two hands in blind and sighted children and adolescents (see however, Bara, 2014, who reported predominantly unimanual explorations in five visually impaired children observed in a joint reading tactile book task). This procedure enlarges the size of the tactile field and this has a positive effect (Loomis et al., 1991), though the benefits could be more complex than this simple extension of the tactile field size (Morash et al., 2013, 2014). The additional use of symmetrical movements may well have been induced by the intrinsic characteristics of the two series of tactile images used in the present experiment, which mainly presented vertical symmetries. Clearly, the specific exploratory procedures employed by blind children to explore objects were related to their performance, and, in particular, to the accuracy of their identifications of these objects.

Using textured images, the proportion of correct semantic identifications increased from 42–43% at 3–6 years of age to 58–62% at 6–8 years of age, regardless of visual status, when only partial information without any geometrical or textural cues was given to the children. By contrast, the age-related improvement in haptic identification was much weaker in previous comparable studies involving the same age range (e.g., Overvliet and Krampe, 2018). In parallel, exploration times increased with age in the sighted children, confirming a finding reported by Overvliet and Krampe (2018), but decreased with age in the blind participants. Moreover, as in Picard et al. (2014), a negative correlation was observed between exploration times and the proportion of correct semantic identifications in the blind group. Taken together, these results suggest that the evolution of haptic identification scores with age reflects improvements in the way children process haptic information, but differently according to their visual status. According to the image-mediation model of haptics developed by Lederman et al. (1990), sighted children would learn to translate haptic information collected during exploration into a visual image that is used to retrieve semantic information, with the result that exploration times increase with age, at least in the early age range involved in the present study. The increase in exploration times could be a consequence of children becoming progressively more able, with age, to keep longer chunks of haptic information in working memory, thereby permitting the elaboration of a visual image that is available for semantic interpretation. As pointed out by Overvliet and Krampe (2018), it is reasonable to think that younger children with reduced working memory capacity more often have recourse to guessing and therefore produce fewer correct naming responses in a shorter time. By contrast, blind children would learn, over the same age period, to process haptic information directly in order to recover shape information, thus leading to a decrease in exploration times due to progressive improvements in the direct semantic interpretation of haptic information.

Other results from our study argue in favor of this view of direct haptic information processing in these children. While the blind children demonstrated similar performances in terms of correct naming responses as their sighted counterparts, they nevertheless produced more correct identifications based on geometrical as well as textural properties, the latter being specific to haptic processing. This bias was observed irrespective of whether these blind children were aged 3-to-6 or 6-to-8 years, and whether or not they received partial information without geometrical or texture cues or full information systematically containing geometrical cues, and occasionally texture cues. It should be noted that the same type of bias was obtained when blind children were asked to verbally define familiar objects, as compared to age-matched sighted children (Vinter et al., 2013). Furthermore, in the blind children, the decrease in exploration times was associated with both a more organized exploration phase (indicated by a more frequent use of bimanual and symmetrical movements) and a better production of correct naming responses. By contrast, in the sighted children, there was no direct connection between exploration times and the use of specific exploratory procedures.

Providing more information about the tactile images in the same way as under natural reading conditions increased the correct semantic identification scores, with overall performance reaching a success rate of 60% at 3–6 years of age and exceeding 80% at 6–8 years of age. This impact of prior information, which has been largely confirmed in the literature (Pathak and Pring, 1989; Heller et al., 1996), was similar in the blind and in the sighted children in our experiment, contrary to the findings reported by Picard et al. (2014), who observed that the visually impaired children made better use of prior category information than their sighted counterparts. However, in the latter study, the comparison was made with a condition in which no information at all was provided. Such a condition is very detrimental to blind participants when performing 2D spatial haptic tasks. This may account for the advantage found in this study in favor of the blind children. It should be noted that, in our experiment, only the blind children made more frequent use of the contour following procedure in the full information condition than in the partial information condition. This result supports the argument that providing these children with prior information helps them to organize their exploratory procedures efficiently, thereby impacting the perceptual processes involved during exploration. This conclusion is opposite to the one defended by Heller et al. (1996), who reported the same effect of prior category information irrespective of whether it was delivered before exploration, or afterward and just before naming. However, their study involved blindfolded sighted adults, not blind individuals. It is likely that the visual mediation used by sighted persons when processing haptic information also permits semantic information to have an impact at a post-perceptual stage, when names are associated with visual images. To conclude regarding the issue of the impact of prior information, we consider that providing such information to blind participants helps them better organize their exploration of the tactile images, with which they are more familiar than sighted individuals. In the latter, this impact probably mainly operates at a post-exploratory stage, when they try to translate haptic information into a visual image as suggested by the image mediation model. At this stage, they may indeed use their advantage in terms of knowledge of the graphic rules governing the building of 2D images.

Finally, our results confirmed the important role played by haptic practice in enabling individuals with vision loss to exhibit satisfactory performance in spatial information processing. The present study showed that the higher the level of regularity of Braille practice and marginally of image tactile practice, the higher the proportion of correct semantic identifications. It also showed that the children with higher levels of regularity of practice with tactile images were less inclined to report geometrical identifications while the contrary was observed in the children with higher levels of regularity of Braille practice. An impact of practice in blind individuals has been demonstrated in several drawing studies involving either children (D’Anguilli and Maggi, 2003; Vinter et al., 2018) or adults (Likova, 2012), as well as in other types of spatial tasks (Dulin and Hatwell, 2006; Postma et al., 2007). Practicing regularly the reading of tactile images probably directs the child to look for cues that support hypotheses about the identity of the objects depicted in these images, thus overshadowing their geometric characteristics. The main aim of the children with frequent tactile image practice would be to discover the meaning of the image they are exploring, making use of shape information of course, but without major interest for it. It could also be that the regular practice of tactile image reading contributes to developing better knowledge of the basic rules of graphic representations, facilitating tactile image recognition. The regular practice of Braille would also orient children to look for the meaning of the explored elements. However, possibly because of the importance of spatial configurations when reading Braille characters, our results suggest that the practice of Braille may also incidentally orient the blind children toward reporting the shapes of the objects explored.

These findings of positive effects of haptic practice in the processing of spatial information points to the major role of education in helping children with vision loss to develop spatial (and, more generally, cognitive) skills to a level of expertise similar to that of age-matched sighted children. Spatial tasks, and especially those involving 2D information, are hard tasks for blind individuals to cope with. However, parents and teachers should be encouraged to confront blind children with this type of task as much as possible and from an early age, supporting them in learning haptic exploration and providing them with cues that facilitate semantic interpretation.

## Data Availability Statement

The datasets generated for this study are available on request to the corresponding author.

## Ethics Statement

Ethical review and approval was not required for the study on human participants in accordance with the local legislation and institutional requirements. Written informed consent to participate in this study was provided by the participants’ legal guardian/next of kin.

## Author Contributions

AV contributed to designing the study, analyzing, interpreting, discussing the data, and writing the manuscript. OO contributed to designing the study, collecting, analyzing, and discussing the data. PM contributed to collecting and analyzing the data.

## Conflict of Interest

The authors declare that the research was conducted in the absence of any commercial or financial relationships that could be construed as a potential conflict of interest.
